# Loss of GltB Inhibits Biofilm Formation and Biocontrol Efficiency of *Bacillus subtilis* Bs916 by Altering the Production of γ-Polyglutamate and Three Lipopeptides

**DOI:** 10.1371/journal.pone.0156247

**Published:** 2016-05-25

**Authors:** Huafei Zhou, Chuping Luo, Xianwen Fang, Yaping Xiang, Xiaoyu Wang, Rongsheng Zhang, Zhiyi Chen

**Affiliations:** 1 College of Plant Protection, Nanjing Agriculture University, Nanjing 210095, China; 2 Institute of Plant Protection, Jiangsu Academy of Agricultural Sciences, Nanjing 210014, China; 3 School of Life Science and Chemical Engineering, Huaiyin Institute of Technology, Huaiyin 223003, China; Loyola University Chicago, UNITED STATES

## Abstract

**Aims:**

This study examined the contribution of GltB on biofilm formation and biocontrol efficiency of *B*. *subtilis* Bs916.

**Methods and Results:**

The *gltB* gene was identified through a biofilm phenotype screen and a bioinformatics analysis of serious biofilm formation defects, and then a *gltB* single knockout mutant was constructed using homologous recombination. This mutant demonstrated severe deficits in biofilm formation and colonisation along with significantly altered production ofγ-polyglutamate (γ-PGA) and three lipopeptide antibiotics (LPs) as measured by a transcriptional analysis of both the wild type *B*. *subtilis* Bs916 and the *gltB* mutant. Consequently, the mutant strain retained almost no antifungal activity against *Rhizoctonia solani* and exhibited decreased biocontrol efficiency against rice sheath blight. Very few *gltB* mutant cells colonised the rice stem, and they exhibited no significant nutrient chemotaxis compared to the wild type *B*. *subtilis* Bs916. The mechanism underlying these deficits in the *gltB* mutant appears to be decreased significantly in production of γ-PGA and a reduction in the production of both bacillomycin L and fengycin. Biofilm restoration of *gltB* mutant by additionγ-PGA in the EM medium demonstrated that biofilm formation was able to restore significantly at 20 g/L.

**Conclusions:**

GltB regulates biofilm formation by altering the production ofγ-PGA, the LPs bacillomycin L and fengcin and influences bacterial colonisation on the rice stem, which consequently leads to poor biocontrol efficiency against rice sheath blight.

**Significance and Impact of Study:**

This is the first report of a key regulatory protein (GltB) that is involved in biofilm regulation and its regulation mechanism and biocontrol efficiency by *B*. *subtilis*.

## Introduction

*Bacillus* biofilms consist of a highly structured extracellular matrix attached to the surface of cells [1–5] that play an important role in the biological control of multiple pathogens [6–8]; for example, the presence of a biofilm can significantly improve the colonisation of *Bacillus amyloliquefaciens* SQR9, and its biocontrol efficiency towards other microbes. Efficient rhizosphere colonisation and biofilm formation enable *Paenibacillus polymyxa* to control crown rot disease [9], while biofilm formation, colonisation, and secretion of surfactin by *B*. *subtilis* 6051 act together to protect plants against pathogenic attack [10].

Although the presence of a biofilm is critical for biological disease control by *Bacillus* species, the mechanisms of biofilm formation, regulation and biocontrol remain unclear [6, 11–12]. The biofilm is primarily composed of a basic skeleton of amyloid fibres that consist of TasA protein, an exopolysaccharide (EPS), and BslA protein [10, 13–15]. In addition to TasA and EPS, γ-polyglutamate (γ-PGA) plays an intricate role in biofilm formation by different *B*. *subtilis* strains. While γ-PGA enhances biofilm formation by *B*. *subtilis* strain RO-FF-1, loss production of γ-PGA had no significant influence on biofilm formation because its function could be substituted by other biofactors [16]. In contrast, a mutant strain of *B*. *amyloliquefaciens* C06 defective in γ-PGA production was unable to form biofilms effectively [17]. Interestingly, however, overproduction of γ-PGA in *B*. *amyloliquefaciens* C06 could not enhance the biofilm formation, but instead inhibited it [18]. Previous studies also showed that many genes are involved in regulating the biofilms of *Bacillus*, including *spo0A*, *spo0H*, and *abrB*, which are needed for EPS and surfactin expression during early sporulation [19]. *spo0A* mutants demonstrate defective cell–cell interactions and cannot form a multicellular biofilm; *abrB* negatively regulates biofilm formation; *sipW* and *yoaW*, which are regulated by AbrB, are also required for normal biofilm formation [20–21]; and *degQ* regulates the production of fengycins and is necessary for normal biofilm formation [22].

In *B*. *subtilis*, lipopeptide antibiotics (LPs) significantly impact biofilm formation [10]. Loss of *ArfB* impairs the production of the LP arthrofactin in *Pseudomonas* sp. MIS38, but enhances biofilm production, although the biofilm is unstable and flat [23–24]. As a very effective biosurfactant, surfactin has been reported to be involved in biofilm formation on solid surfaces [9, 25–26]. Surfactin is also required for biofilm formation on liquid cultures by *B*. *subtilis* strain A1/3 [27]. Other studies have suggested that surfactin contributes to biofilm formation by secreting a signalling molecule for KinC activation of the Spo0A pathway [28–29]. The LP bacillomycin D contributes to biocontrol activity and to biofilm formation in *B*. *amyloliquefaciens* SQR9 [30]. We have previously used high performance liquid chromatography-mass spectroscopy (HPLC-MS) to identify three LPs (bacillomycin L, surfactin, and fengycin) secreted by *B*. *subtilis* Bs916 [31–33], and determine that both bacillomycin L and surfactin were necessary for biofilm formation by this strain [34–35]. While fengycin had no influence on biofilm formation, it played an important role in the biocontrol efficiency of *B*. *subtilis* Bs916 because of its antifungal properties [32].

In this study, we report a critical regulatory protein in *B*. *subtilis* Bs916, named GltB, identified in a screen for altered biofilm phenotypes. After mutating the *gltB* gene in *B*. *subtilis* Bs916 by homologous recombination, the resulting biofilm was weak, thin and structurally distinct from that of the wild-type (WT) *B*. *subtilis*. Transcriptomic analysis revealed the production of γ-PGA and the three LPs, bacillomycin L, surfactin, and fengycin was closely related to biofilm formation; production of these molecules was also investigated in detail by HPLC-MS. Additionally, the antifungal activity, colonization ability and biocontrol efficiency of the *gltB* mutant against *Rhizoctonia solani* were also investigated. GltB markedly affected the production of γ-PGA and altered the secretion of the LPs surfactin, bacillomycin L and fengycin, which resulted in poor biofilm formation, colonisation and biocontrol efficiency by *B*. *subtilis* Bs916.

## Materials and Methods

### Strains and culture conditions

The strains and plasmids used in this study are listed in Table 1. The *B*. *subtilis* Bs916 and the *gltB* mutant were cultured in Luria–Bertani (LB) broth medium [36] (10 g/L tryptone, 5 g/L yeast extract, 10 g/L NaCl) at 37°C under shaking at 200 rpm. *Escherichia coli* DH5α cultured in LB broth medium was used to produce mutant vectors for eventual transformation into *B*. *subtilis* Bs916. *R*. *solani* was cultured at 28°C on potato dextrose agar (PDA) medium (200 g/L potato infusion, 20 g/L glucose, and 20 g/L agar at pH 7.0). Biofilm formation of both *B*. *subtilis* Bs916 and the *gltB* mutant was assessed using MSgg medium [19] [5 mM potassium phosphate (pH 7), 100 mM Mops (pH 7), 2 mM MgCl_2_, 700 μM CaCl_2_, 50 μM MnCl_2_, 50 μM FeCl_3_, 1 μM ZnCl_2_, 2 μM thiamine, 0.5% glycerol, 0.5% glutamate, 50 μg/mL tryptophan, 50 μg/mL phenylalanine] and EM medium [(1L, PH 7.4): 20g L- glutamate, 12g citric acid, 80g glycerine, 7g ammonium chloride, 0.5g magnesium sulfate heptahydrate, 0.5g dipotassium phosphate, 0.15g calcium chloride dehydrate, 40mg ferric chloride hexahydrate, and 148mg manganese sulphate monohydrate] [37]. If necessary, antibiotics were added at the following concentrations: 100 mg/L spectinomycin, 100 mg/L ampicillin, and 5 mg/L chloramphenicol.

**Table 1:** Bacterial strains and plasmids used in this study.

Strain/plasmid	Description	Source or reference
Strains		
*B*. *subtilis* Bs916	Wild type strain	CGMCC* No. 0808
Δ*gltB*	Δ*gltB*::Spec^r^*, Bs916 derivative	This study
ΔBs916-gfp	ΔBs916-gfp::Cm^r^, Bs916 tagged with green fluorescent protein	This study
Δ*gltB*-gfp	Δ*gltB*:: Spec^r^*, Δ*gltB* tagged with green fluorescent protein	This study
Plasmids		
pUC19	Cloning vector, Ap^r^	TakaRa U07650, Dalian, China
pDG1728	Cloning vector, Amp^r^* Spec^r^*	BGSC No. ECE114
pUCSpec	pUC19 carrying spectinomycin cassette from pDG1728	This study
pUCSpec-gltB	pUCSpec carrying 741 bp fragment *gltB*	This study
pRp22-gfp		This study

*Amp^*r*^: ampicillin resistance, *Spec*^*r*^: spectinomycin resistance, *CGMCC*: China General Microbiological Culture Collection Centre, Beijing, China.

### *B*. *subtilis* Bs916 mutant library construction and identification of the *gltB* gene

This method has been previously described in Scientia Agricultura Sinica [38]. Briefly, a mutant library of *B*. *subtilis* Bs916 was created using the plasmid pMarA carrying the transposon TnYLB-1, which resulted in random insertion at chromosomal loci. Single colonies from this mutant library were inoculated into 4 mL of LB medium containing spectinomycin to select for mutants and shaken for 12 h at 37°C, and single colonies of the control strain *B*. *subtilis* Bs916 were also cultured without spectinomycin. A 200 μl aliquot of the culture was inoculated into 4 mL of MSgg medium and was grown in a stationary setting for 24 h at 37°C. Each sample was repeated three times. A visual inspection revealed whether there was an obvious change in biofilm formation between the mutant and the WT strains. After screening 5000 mutants, we identified the *gltB* mutant as having significant deficiencies, and we detected only a single insertion site by Southern Blot.

### Construction of the Δ*gltB* mutant and the addition of GFP tags

A spectinomycin resistance gene and its promoter were obtained from plasmid pDG1728 using the primers SpecF (5'-TTTGGATCCCTGCAGCCCTGGCGAATG-3') and SpecR (5'-TTTGAATTCAGATCCCCCTATGCAAGG-3'; *Bam*HI and *Eco*RI restriction sites, respectively, are underlined). The complete spectinomycin cassette was isolated via digestion with *Bam*HI and *Eco*RI and was cloned into the plasmid pUC19; the resulting plasmid was named pUCSpec. A 741-bp PCR product of *gltB* was cloned from the genome of WT *B*. *subtilis* 916 using the primers gltBF (5'-TTTAAGCTTTAACTGTCGACTTCGCGCG-3') and gltBR (5'-TTTGGATCCTGATGCCGTCATTTTGTGC-3'; *Hind*III and *Bam*HI restriction sites, respectively, are underlined). Next, this PCR product was inserted into pUCSpec to create the pUCSpec-gltB plasmid, and the Δ*gltB* mutant was obtained by transforming the pUCSpec-gltB plasmid into WT *B*. *subtili*s 916. Finally, the positive emission green fluorescent protein (GFP) strains ΔBs916-gfp and Δ*gltB*-gfp were constructed by transforming the plasmid pRp22-gfp into both the WT *B*. *subtilis* 916 and the Δ*gltB* mutant.

### Biofilm formation by WT *B*. *subtilis* Bs916 and the Δ*gltB* mutant

To assess biofilm formation, a single colony of either WT *B*. *subtilis* 916 or the *gltB* mutant was inoculated into 4 mL of LB medium (containing spectinomycin only for mutants) and shaken overnight at 37°C. Approximately 1 mL of this culture was then inoculated into 50 mL of LB medium and shaken for 12 h at 37°C. Subsequently, 200 μl of this culture was inoculated into 4 mL of MSgg containing 20 μg/mL Congo Red and 10 μg/ml Coomassie brilliant blue in 12-well microtiter plates at 37°C for 24 h [13]. Finally, we transferred the biofilm of each mutant into 2 mL tubes, placed the tubes in a drying oven at 37°C, and weighed each sample. Each experiment was repeated three times.

### Analysis of genomic transcript levels of WT *B*. *subtilis* Bs916 and the Δ*gltB* mutant using Illumina Hiseq^TM^2500

Total biofilm RNA of both WT *B*. *subtilis* 916 and the Δ*gltB* mutant cultured in a static MSgg medium (4 mL in 12-well microtiter plates) at 37°C for 24 h was extracted using an RNAprep pure Cell/Bacteria Kit. Qualified RNA was obtained according to the 2100 Bioanalyzer test, and then 10 μg of total RNA was digested using 5U DNaseI (Takara, Japan) for 30 min at 37°C. The RNA was then purified using an RNeasy MinElute Cleanup Kit (Qiagen, Germany) and eluted with 15 μl RNase-free water. All rRNA was removed using a Ribo-Zero™ Magnetic Kit (Gram-negative or Gram-positive bacteria; Epicentre, USA) in which the total reaction volume was brought to 40 μl by adding Ribo-Zero Reaction Buffer and Ribo-Zero rRNA Removal Solution, and the reaction was carried out at 68°C for 10 min and then at room temperature for 5 min. Treated RNA was added into pre-washed beads, mixed well, and incubated at room temperature for 5 min. Next, it was moved to 50°C for 5 min, immediately transferred to a magnetic rack for 1 min, and brought to a volume of 180 μl with water. Next, 3 M sodium acetate, glycogen (10 mg/ml), and 600 μl ethanol were added to the supernatant, and the mixture was placed at -20°C for at least one hour. The rRNA-depleted RNA was then isolated via centrifugation, and the precipitate was dissolved in water. A 100-ng aliquot of rRNA-depleted RNA was used to construct a cDNA library with the NEB Next ^®^ Ultra ^TM^ Directional RNA Library Prep Kit for Illumina (NEB, USA). The cDNA library was checked via Qubit fluorometric quantitation, 2% agarose gel electrophoresis, and a high-sensitivity DNA chip. A cluster generation of the cDNA library (10 ng) was executed in cBot using the TruSeq PE Cluster Kit (Illumine, USA) and sequenced using a Illumina Hiseq^TM^2500.

Linker sequences that contained low quality clean reads were removed by fastx_clipper, and unwanted, low quality (>20 bases) reads from 3’- 5’ were removed using a FASTQ Quality filter (FASTX-Toolkit, v0.0.13, http://hannonlab.cshl.edu/fastx_toolkit/), thereby deleting all clean reads less than 50 bp. The quality of the sequencing data was assessed using Fastqc software (v0.10.0). Differential gene expression analysis was completed by mapping clean reads (Bowtie 2, v2.1.0) and using the MA-plot-based method with the random sampling model (version 1.20.0). The overall expression levels of each gene were calculated both in the WT Bs916 and Δ*gltB* mutant strains. When differentially expressed genes satisfied several conditions, including a fold change > 2, an FDR(q value) < 0.001, and at least one sample with an RPKM > 20, their expression was considered significantly altered, and these genes were set aside for further analysis.

### Antifungal properties of WT *B*. *subtilis* Bs916 and the Δ*gltB* mutant against *R*. *solani*

Mycelial plugs of *R*. *solani* (7 mm diameter) cultured in PDA medium for 3 d were placed in the centre of LB plates, and approximately 2 μl of cultured WT *B*. *subtilis* 916 or *gltB* mutant was dropped onto the surface of the circular filter paper (6 mm diameter) in an even distribution around the mycelial plugs. The plates were sealed with Parafilm and incubated at 28°C. The antibacterial bandwidth was measured after 24–72 h. Each experiment was repeated three times.

### Detection of bacterial colonisation in the rice plant via confocal microscopy and determination of its biocontrol efficiency against rice sheath blight

The bacterial cakes (6 mm diameter) of *R*. *solani* cultured for 3 d in solid PDA medium were inoculated into 100 mL of liquid PDA medium with 200 matchstick without commelina. The cakes were incubated in a 500-mL flask without shaking at 28°C for one week. Thirty seeds of rice cultivar were soaked in water for 24 h and then sown in the nursery containing sterile organic soil. At 5 d before rice heading, 10 matchsticks of *R*. *solani* were inoculated into 2–3 cm rice leaves of each rice plant. Approximately 20 mL of broth (cultured for 48 h) containing WT *B*. *subtilis* Bs916-gfp or the Δ*gltB*-gfp mutant were evenly sprayed into the rice plant marked by a matchstick. From 0–15 d after the initial inoculation, the rice leaf marked by a matchstick was clipped and captured with a 40× objective microscope using a confocal microscope (Carl Zeiss LSM710, Germany). To determine the biocontrol efficiency against rice sheath blight, the lesion area length of the WT ΔBs916-gfp and the Δ*gltB*-gfp mutant were scored. Each treatment group contained eight individual pots.

### Analysis of bacillomycin L, surfactin, and fengycin levels produced by WT *B*. *subtilis* Bs916 and the Δ*gltB* mutant

A single colony of WT *B*. *subtilis* 916 or the *gltB* mutant was inoculated into 4 mL of LB medium (containing spectinomycin for mutants) and shaken overnight at 37°C, and then approximately 1 mL of the resulting culture was inoculated into 50 mL of LB medium and shaken for 48 h at 37°C. The supernatant of the WT *B*. *subtilis* 916 and the Δ*gltB* mutant cultures were isolated via centrifugation at 9,000 rpm for 20 min and then precipitated by adding an appropriate amount of hydrochloric acid to pH 2.0. The lipopeptide precipitates were collected via centrifugation at 12,000 rpm for 30 min, dried, and extracted using 2 mL of methanol before being passed through a 0.22 μm filter and then successfully separated using an Agilent 1200 high-performance liquid chromatography system with a photodiode array (HPLC-PDA) (Agilent Technologies, Santa Clara, USA) attached to a reversed-phase C18 column (5 μm, 4 mm × 250 mm; Merck, Frankfurt, Germany). The run was performed at a flow rate of 0.5 mL/min, a detection wavelength of 210 nm, and a gradient of solvents A (60% water containing 0.5% trifluoroacetic acid) and B (40% acetonitrile containing 0.5% trifluoroacetic acid) for bacillomycin L, A (20% water containing 0.5% trifluoroacetic acid) and B (80% acetonitrile containing 0.5% trifluoroacetic acid) for surfactin, and A (50% water containing 0.5% trifluoroacetic acid) and B (50% acetonitrile containing 0.5% trifluoroacetic acid) for fengycin [34]. The lipopeptide precipitates were further analysed using an Agilent 6410 Triple Quadrupole liquid chromatography/mass spectrometer (LC/MS) (Agilent Technologies, Santa Clara, CA, USA) in positive ion electrospray mode.

### Determination of γ-polyglutamate (γ-PGA) and glutamate content of WT *B*. *subtilis* Bs916 and the Δ*gltB* mutant in the process of biofilm formation

To assess the glutamate content of WT *B*. *subtilis* Bs916 and the Δ*gltB* mutant in the process of biofilm formation in the EM medium, the glutamic acid derivative reagent was obtained containing the following ingredients: 0.1g o-phthaldehyde (OPA), 2.5 mL methanol, 100 μL 2-mercaptoethanol, 22.4 mL 0.4 mol/L sodium borate buffer (PH 9.5). 1 g/L standard L-glutamate was prepared. 1 mL bacteria of biofilm below in 12-well microtiter plates was collected via centrifugation at 12,000 rpm for 5 min. 100 μL standard L-glutamate, supernatant, and EM medium were combined with 100 μL glutamic acid derivative reagent for 90 s respectively. Derivatized samples of the *B*. *subtilis* Bs916, Δ*gltB* mutant, standard L-glutamate, and EM medium were filtered by 0.22 μm membrane. In addition to standard L-glutamate and double dilution to *B*. *subtilis* Bs916, all treatments were diluted tenfold for detection by using an Agilent 1200 high-performance liquid chromatography system with a photodiode array (HPLC-PDA) (Agilent Technologies, Santa Clara, USA) attached to a reversed-phase C18 column (5 μm, 4 mm × 250 mm; Merck, Frankfurt, Germany). The run was performed via the gradient elution (Table 2).

**Table 2:** Program of gradient elution.

Min	Phosphate buffer (%)	Methanol (%)	Flow rate (mL/min)
0	83	17	1.2
3	62	38	1.2
3.2	60	40	1
12	60	40	1
13	83	17	1.2
15	83	17	1.2

Mobile phase A: Methanol, B: 10 mmol/L Phosphate buffer (PH 6.85). Detection wavelength: 338 nm. Column temperature: 30°C. Injection volume: 10μL.

Method of acid hydrolysis was used to assess theγ-PGA content of WT *B*. *subtilis* Bs916 and the Δ*gltB* mutant in the process of biofilm formation. 200 μL overnight LB medium of the *B*. *subtilis* Bs916 and the Δ*gltB* mutant was inoculated into 4 mL of EM medium in 12-well microtiter plates at 37°C for 24 h. 4 mL bacteria of biofilm below in 12-well microtiter plates was collected via centrifugation at 12,000 rpm for 5 min. The supernatant was precipitated with three times ethanol for 12 h respectively. The precipitation was collected by 12000 rpm centrifugation for 30 min, and was dissolved with 4 mL distilled water. 2 mL 6N hydrochloric acid was added into 2 mL this dissolving liquid, then was allowed to stand at 110°C overnight without oxygen. Method of glutamic acid detection was same as above.

### Biofilm restoration of the Δ*gltB* mutant by addition γ-PGA in the medium

Theγ-PGA added to the EM medium to the final concentration at 10 g/L, 20 g/L, and 30 g/L respectively were used to restore the biofim formation of Δ*gltB* mutant. 200 μL overnight culture of *B*. *subtilis* Bs916 or Δ*gltB* mutant were inoculated in 4 mL EM medium with or without addition γ-PGA respectively, and stand at 37°C for 24 h to form biofilm. Polyaspartic acid was added as a control. Each treatment has three duplicates and repeated three times. To further test biofilm formation of Δ*gltB* mutant, grew next to the WT *B*. *subtilis* Bs916 for 72 h.

## Results

### Detection of *gltB* transposon insertion by alterations in biofilm formation

By screening 5000 mutants from the random insertion mutant library of the WT *B*. *subtilis* 916 by biofilm phenotype variety, we identified eight mutants that resulted in major changes in biofilm formation. In one mutant that contained the *gltB* transposon insertion, a significant and powerful defect in biofilm formation was observed compared to the WT.

**Fig 1:**
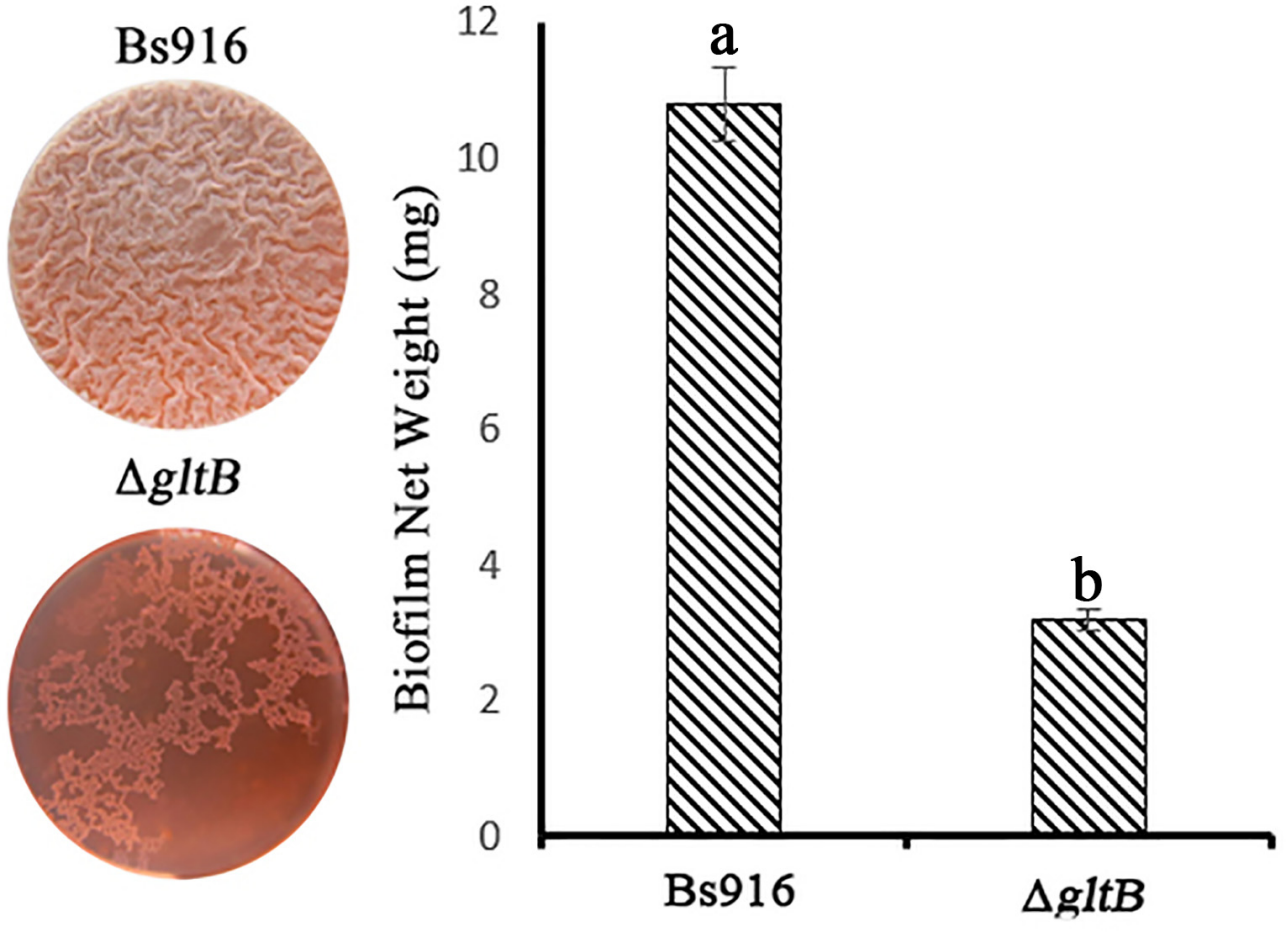
Changes in biofilm formation due to the *B*. *subtilis* Bs916 and Δ*gltB* mutants in MSgg culture medium with 20 μg/mL Congo Red and 10 μg/mL Coomassie brilliant blue. (1) *B*. *subtilis* Bs916 biofilm was dense and solid with clear lines. In contrast, the Δ*gltB* mutant formed an uneven biofilm with an irregular shape. (2) The net weight of the Δ*gltB* mutant biofilm was more than three times less than the net weight of the WT *B*. *subtilis* Bs916 biofilm.

By disrupting the *gltB* gene through homologous recombination, we successfully constructed a single knockout (Δ*gltB* mutant). Even after continuous culture in MSgg medium for 24 h, severe deficits in biofilm formation persisted. The Δ*gltB* mutant biofilm was thin and fragile and consisted only of a planar structure, as opposed to the clear three-dimensional structure observed in the WT *B*. *subtilis* Bs916 biofilm (Fig 1). The dry weight of the Δ*gltB* mutant was calculated as only one-third of the WT *B*. *subtilis* Bs916. The morphology of the mutant colonies also differed, with only a thin planar structure observed in the Δ*gltB* mutant compared with an obvious red bulge of the WT *B*. *subtilis* Bs916 (S1 Fig). This phenotype remained stable over time, suggesting that the *gltB* gene plays an important role in the proper formation of a *B*. *subtilis* Bs916 biofilm.

### Analysis of differentially expressed genes in WT *B*. *subtilis* Bs916 and Δ*gltB* mutant

**Fig 2:**
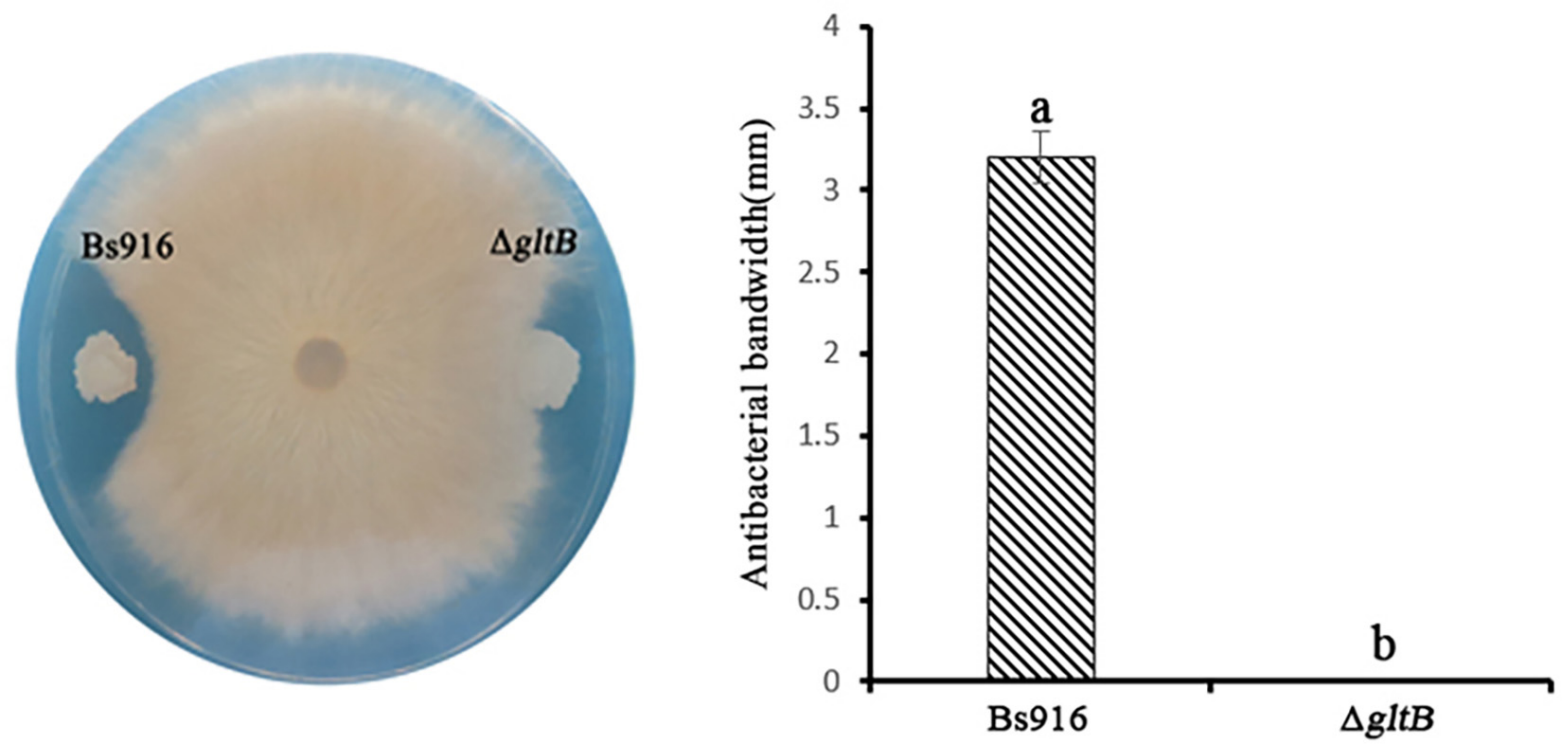
Differences in antibacterial activity against *R*. *solani* between WT *B*. *subtilis* Bs916 and the Δ*gltB* mutant. The Δ*gltB* mutant completely lost its antibacterial activity compared to the WT *B*. *subtilis* Bs916.

Differentially expressed genes were identified by analysing gene transcription (Table 3), and the levels of *bmy* and *fen* were significantly lower in the Δ*gltB* mutant (approximately 5% and 7% of WT levels, respectively). The *bmy* and *fen* genes code for the biosynthesis of LPs bacillomycin L and fengycin, respectively. In contrast, the Δ*gltB* mutant exhibited approximately a five-fold increase in *srfA* transcription compared to WT *B*. *subtilis* Bs916. Another significant increase was noted in *dhb* transcription. This gene, which is responsible for the biosynthesis of the non-lipopeptide antibiotic bacillibactin, increased by 121-fold in the Δ*gltB* mutant and it may be a compensatory mechanism for the lack of bacillomycin L and fengycin; however, this change in expression had no impact on antibacterial activity or biocontrol efficiency against *R*. *solani* (Fig 2). The *ykuP*-*N*-*O* gene cluster is identified as responsible for the flavodoxin synthesis, and mainly use to inhibit bacterial, especially *Staphylococcus aureus*, *Streptococcus*, *Diphtheria*, etc, and no significant effect on fungal pathogens. The *ybdZ* is generally considered for MbtH-like protein synthesis, such as the peptide siderophore coelichelin and the calcium-dependent peptide antibiotic (CDA) [39], and its function is similar to bacillibactin. The *yktc1* encodes 4-aminobutyrate aminotransferase synthesis, its main function is responsible for the conversion between amino acids and keto acid, and may be included in the metabolic pathways of glutamate toγ-PGA. Although they are necessary raw materials for biofilm formation, the levels of TasA and EPS did not significantly change in the Δ*gltB* mutant. Another important raw material for the synthesis of a biofilm, the levels of CapB (YwsC) was only one fifth of the WT *B*. *subtilis* Bs916, which was considered to be necessary for γ-PGA production [40]. It may be due to a lack of γ-PGA for bad biofilm formation in the Δ*gltB* mutant. Furthermore, the expression levels of several previously identified negative regulatory genes of biofilm formation, including *abrB* and *sinR*, were doubled in the Δ*gltB* mutant [20, 41–43]. In contrast, the expression of the positive regulatory genes *spo0A* was somewhat higher in the Δ*gltB* mutant, but the increase was less than that of the negative regulatory genes. These changes in gene transcription suggested that *bmy*, *srfA*, *fen*, and *capB* were all regulated by *gltB*, and their altered expression results in the significant deficit in biofilm formation observed in the mutant version of *B*. *subtilis* Bs916.

**Table 3:** Analysis of differentially expressed genes in wild type *B*. *subtilis* Bs916 and the Δ*gltB* mutant.

Gene	Relative expression level		Function
	*B*. *subtilis* Bs916	Δ*gltB*	
*yktc1bmyA-D*	11	0.050.05	4-aminobutyrate aminotransferaseBacillomycin L biosynthesis
*fenA-E*	1	0.07	Fengycin biosynthesis
*hpr*	1	0.08	HTH-type transcriptional regulator
*tcyJ-N*	1	0.09	Sulphur containing amino acid ABC
*senN*	1	0.11	SenN transcriptional regulator
*appA–D*	1	0.12	Periplasmic oligopeptide-binding protein
*nprE*	1	0.12	Extracellular neutral metalloprotease
*tasA*	1	0.67	Substrate for biofilm formation
*sipW-yqxM*	1	1.30	Type I signal peptidase protein for biofilm formation
*kinA-D*	1	1.63	Two-component sensor histidine kinase
*spo0A*	1	1.65	Response regulator
*degQ*	1	0.96	Pleiotropic regulator
*sinR*	1	2.03	Master regulator of biofilm formation
*epsA-B*	1	2.15	Substrate for biofilm formation
*abrB*	1	2.23	Transcriptional regulator for transition state genes
*srfAA-D*	1	5.09	Surfactin biosynthesis
*ybdZ*	1	112.80	MbtH-like protein
*dhbA-E*	1	121.12	Bacillibactin biosynthesis
*ykuP-N-O*	1	121.23	Short-chain flavodoxin
*cfr*	1	247.70	Ribosomal RNA large subunit methyltransferase Cfr
*capB*	1	0.21	γ-PGA synthesis

### WT *B*. *subtilis* Bs916 had higher antibacterial activity and greater biocontrol efficiency against *R*. *solani*

Basically no antibacterial activity was displayed by the Δ*gltB* mutant compared to a 5-mm antibacterial bandwidth of WT *B*. *subtilis* Bs916 (Fig 2). Unsurprisingly, the Δ*gltB* mutant also demonstrated poor biocontrol efficiency against rice sheath blight. WT *B*. *subtilis* 916 demonstrated 65.7% biocontrol efficiency on the third day when lesions on the rice stem were just appearing, which dropped to 36% by the fifteenth day. In contrast, the Δ*gltB* mutant only achieved 11.4% biocontrol efficiency on the third day, which is approximately 17% of the value for the WT. As the onset of rice sheath blight progressed, the Δ*gltB* mutant could only achieve a biocontrol efficiency of 1.7% by the fifteenth day, which was significantly lower than the 36% of the WT *B*. *subtilis* 916 at the same time point (Table 4). Taken together, these results suggest that the Δ*gltB* mutant exerts little to no biocontrol effect against *R*. *solani*.

**Table 4:** Biocontrol of rice sheath blight in pot cultures of WT *B*. *subtilis* 916 and the Δ*gltB* mutant strain.*

Treatment	3 d		6 d		9 d		12 d		15 d	
	Lesion size (cm^2^)	Controlefficiency (%)	Lesion size (cm^2^)	Controlefficiency (%)	Lesion size (cm^2^)	Controlefficiency (%)	Lesion size (cm^2^)	Controlefficiency (%)	Lesion size (cm^2^)	Controlefficiency (%)
Bs916	1.2(0.3)^b^	65.7^a^	3.9(0.4)^c^	57.6^a^	8.2(1.1)^c^	51.2^a^	16.5(1.9)^c^	43.0^a^	26.5(2.7)^c^	36.0^a^
Δ*gltB*	3.1(0.2)^a^	11.4^b^	8.5(0.6)^b^	7.6^b^	16.1(1.2)^b^	4.2^b^	27.7(2.0)^b^	4.1^b^	39.8(3.0)^b^	3.9^b^
CK	3.5(0.2)^a^		9.2(0.5)^a^		16.8(0.9)^a^		28.9(2.2)^a^		41.4(2.9)^a^	

*Data are the average of five pots ± the standard deviation of three independent experiments with five pots. a, b, and c mean in the same column with different letters are significantly different (P<0.05) according to Duncan’s multiple range tests.

### Poor colonisation by the Δ*gltB* mutant

**Fig 3:**
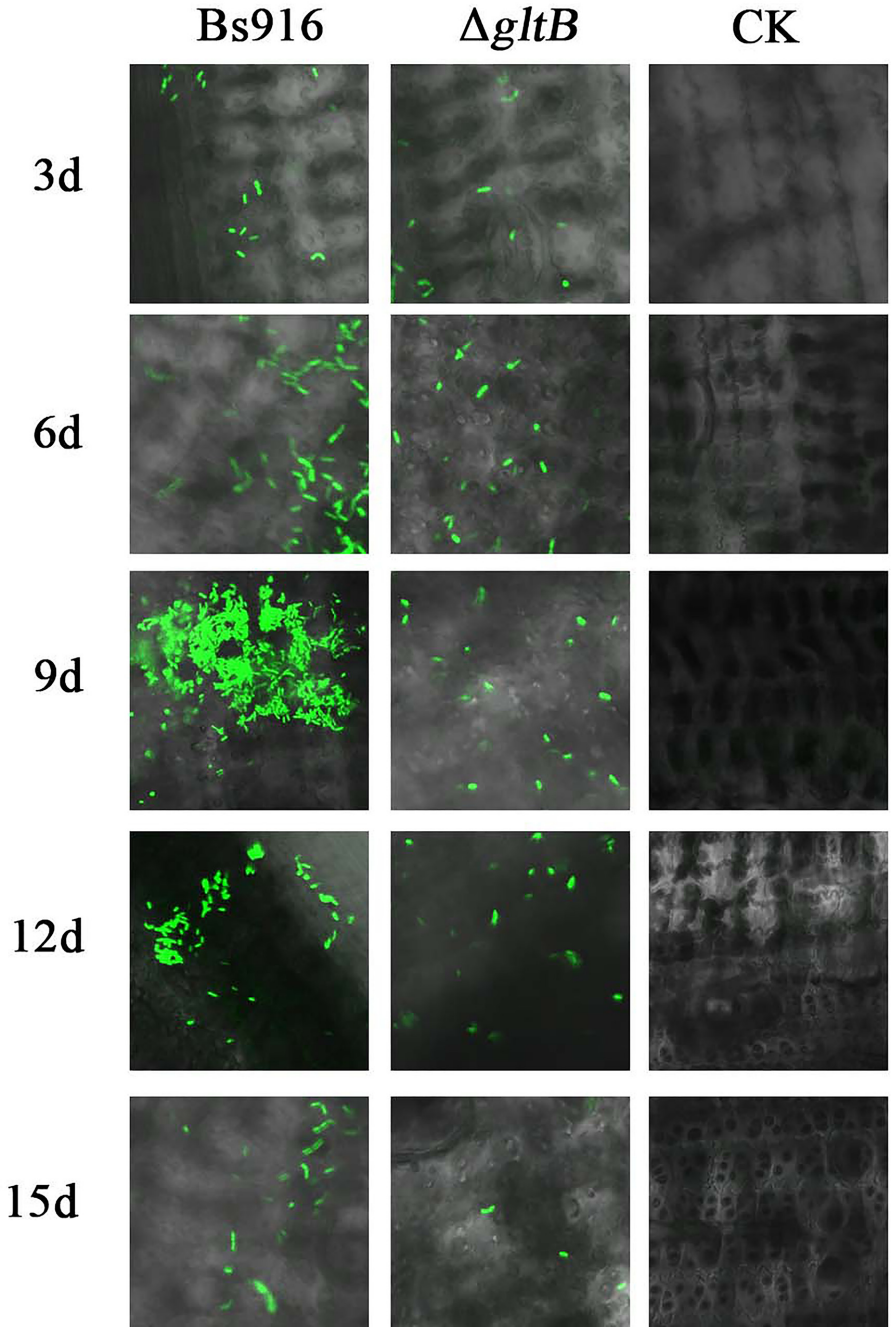
Colonisation of the rice plant against rice sheath blight. Over time, the number of both WT *B*. *subtilis* Bs916 and Δ*gltB* mutant cells initially increased and then began to decrease. However, although WT *B*. *subtilis* Bs916 could produce normal clusters of cells, the Δ*gltB* mutant also demonstrated this same trend. By the last time point (i.e., 15 d), there were very few cells in either the WT *B*. *subtilis* Bs916 or the Δ*gltB* mutant, and the clustering of WT *B*. *subtilis* Bs916’s had disappeared.

The capacity for colonisation is a key factor in the prevention and treatment of fungal diseases because colonisation on host plants is closely related to biofilm formation in that strong colonisation often leads to good biofilm formation. Previous studies have suggested that mucoid mutants of the biocontrol strain *Pseudomonas fluorescens* CHA0, which have enhanced biofilm formation, also display significantly enhanced colonisation in carrot roots [44]. Using a GFP-labelling technique in this study, we assessed the colonisation abilities of the Δ*gltB* mutant and the WT *B*. *subtilis* Bs916 on a rice stem that had been inoculated with *R*. *solani* (Fig 3). After six days, the Δ*gltB* mutant demonstrated poor colonisation, not only because there were fewer bacterial cells but also because the mutant cells lacked significant nutrient chemotaxis. This was especially clear by day nine, when the WT *B*. *subtilis* Bs916 cells displayed strong green fluorescence and an obvious clustering effect, whereas only small scattering of green fluorescence was observed in the mutant cells via confocal microscopy. Although the overall number of bacteria began to decline, this clustering effect was still clearly observed in the WT *B*. *subtilis* Bs916 through day twelve. Similar to day nine, only a small scattering of green fluorescence was observed in the Δ*gltB* mutant. By day fifteen, only a few bacterial cells were left in both the WT *B*. *subtilis* Bs916 and the Δ*gltB* mutant. The results suggest that the *gltB* gene is essential for the regulation of bacterial colonisation in *B*. *subtilis* Bs916, which may also be influenced by the strength of the biofilm formation and its γ-PGA production [17].

### Disrupting *gltB* gene expression significantly altered the production of lipopeptide antibiotics bacillomycin L, surfactin, and fengycin in WT *B*. *subtilis* Bs916

**Fig 4:**
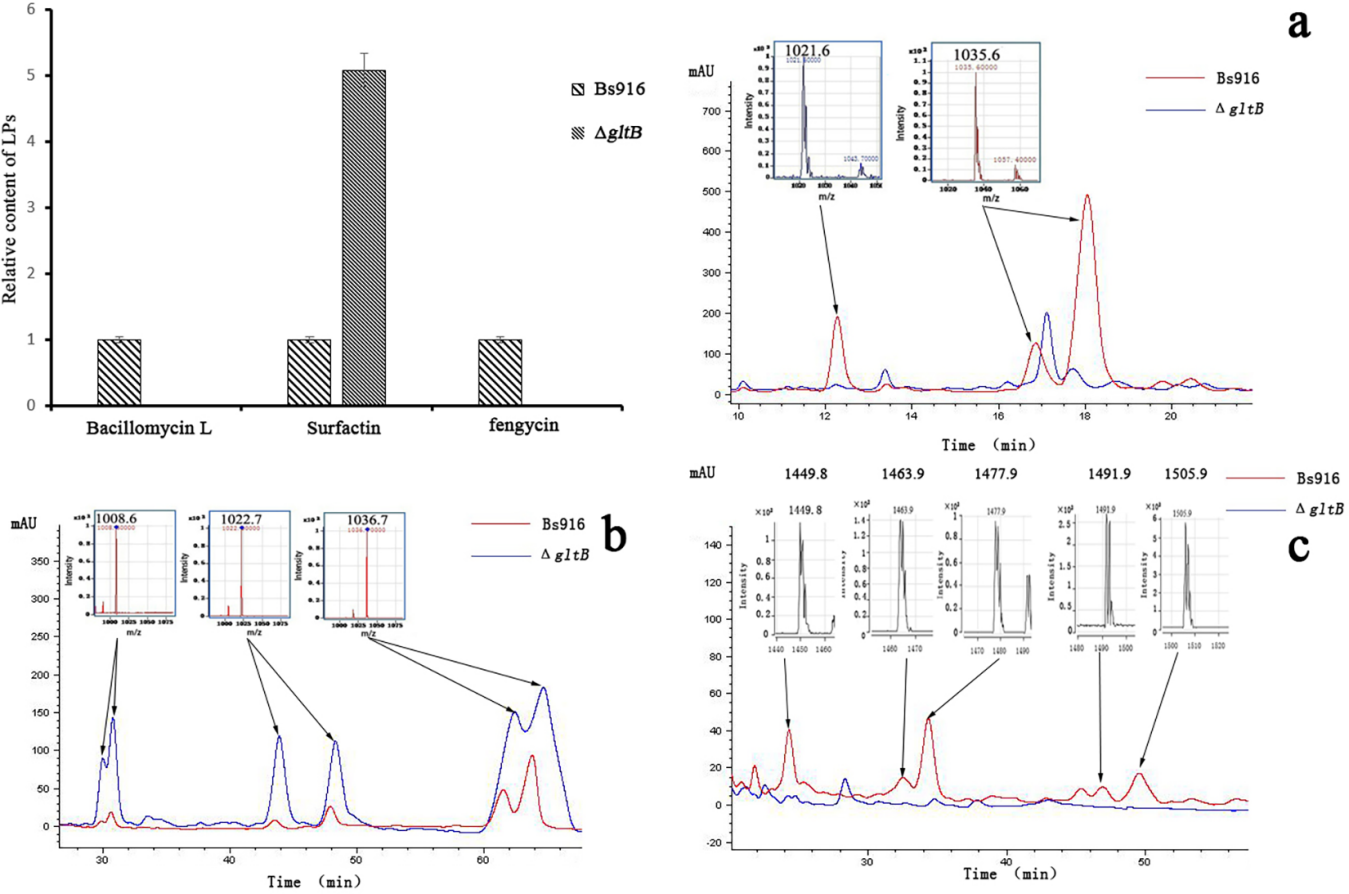
**Antibiotic secretion of bacillomycin L (a), fengycin (c), and surfactin (b).** The Δ*gltB* mutant no longer secreted bacillomycin L or fengycin. In contrast, the Δ*gltB* mutant produced significantly higher (approximately five times higher) levels of surfactin compared to the WT *B*. *subtilis* Bs916.

LPs play a major role in the control of rice fungal diseases such as rice sheath blight and rice blast. Based on our previous data and a comparison of our MS molecular weight data with other *B*. *subtilis* mass data, six peaks of surfactin, three peaks of bacillomycin L, and five peaks of fengycin were identified in the WT *B*. *subtilis* Bs916 by HPLC-MS [45–47] (Fig 4). However, neither bacillomycin L nor fengycin were detected when we analysed the Δ*gltB* mutant supernatants via HPLC. Surprisingly, five times the surfactin was detected in the Δ*gltB* mutant compared to the WT *B*. *subtilis* Bs916 (Fig 4). Approximately 19.7 mg/L and 22.8 mg/L of bacillomycin L and surfactin, respectively, were produced in *B*. *subtilis* Bs916 after 36 h of cultivation in LB medium [34]. Although the production of fengycin in *B*. *subtilis* Bs916 was lower (3 mg/L), it still has a strong ability to inhibit fungal growth [32]. Although the surfactin production of the Δ*gltB* mutant was five times higher than WT *B*. *subtilis* Bs916, the increased production of surfactin could not compensate for the reduction in bacillomycin L and fengycin production, and therefore the ability of the Δ*gltB* mutant to inhibit the growth of *R*. *solani* was significantly reduced almost to zero. Taken together, these results suggest that *gltB* plays a key role in regulating the production of LPs, particularly bacillomycin L and fengycin, which play key roles in controlling rice sheath blight.

### Glutamate consumption and γ-PGA production of Δ*gltB* mutant and WT *B*. *subtilis* Bs916

**Fig 5:**
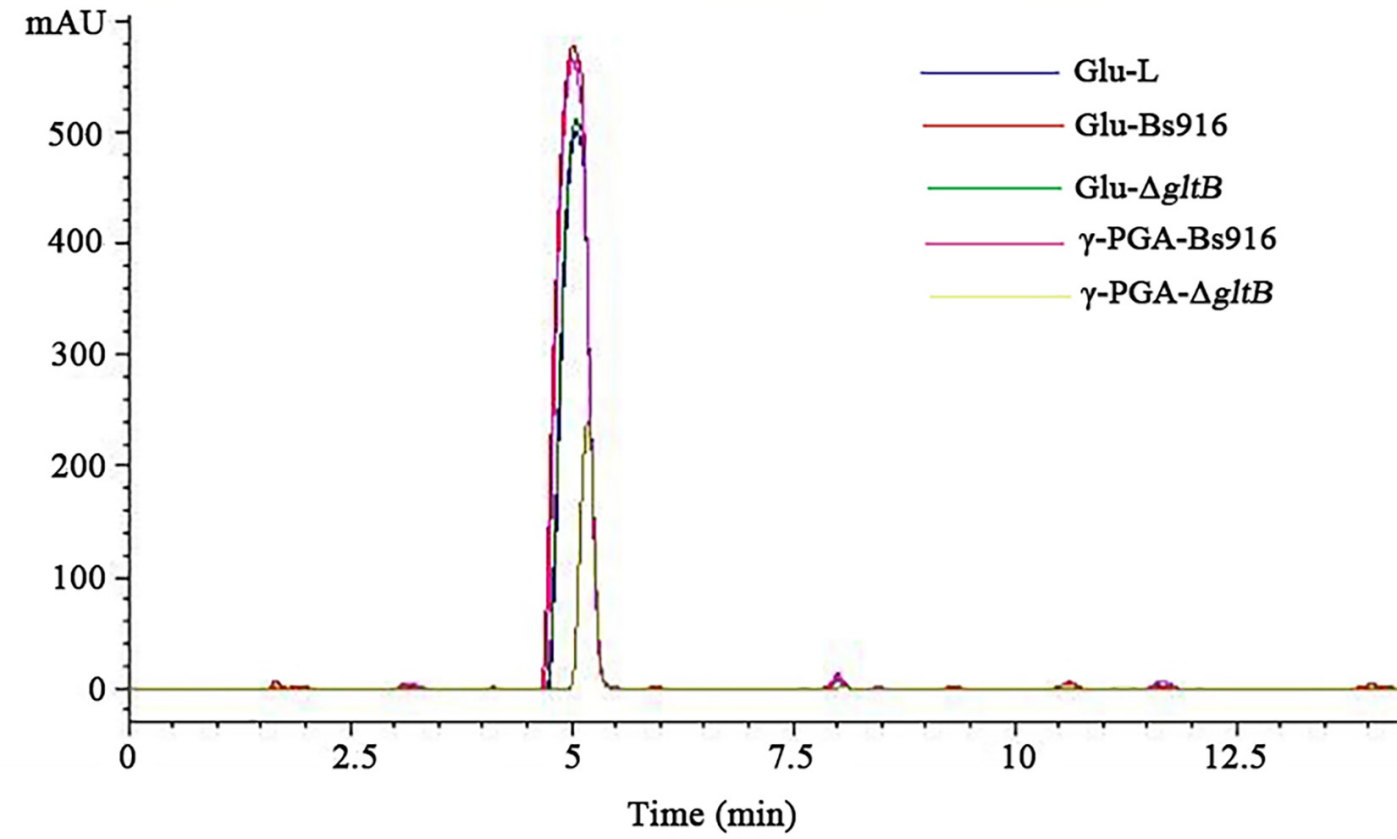
γ-PGA and glutamate content of WT *B*. *subtilis* Bs916 and the Δ*gltB* mutant in the process of biofilm formation. For glutamate detection, in addition to standard L-glutamate and double dilution to *B*. *subtilis* Bs916, all treatments were diluted tenfold for detection. For γ-PGA detection, all treatments were also diluted tenfold for detection.

As the Fig 5 and Table 5 shown, the WT *B*. *subtilis* Bs916 was able to consume glutamate efficiently and decrease the concentration of glutamate from 20 g/L to the 2.68 g/L after 24 h culture. Inversely, the Δ*gltB* mutant was unable to take advantage of glutamate as efficiently as the WT *B*. *subtilis* Bs916 and only decreased to 10.41 g/L. As expect, the production of γ-PGA of *B*. *subtilis* Bs916 was able to reach 13.46 g/L after 24 culture. However, the production of γ-PGA of Δ*gltB* mutant was only reach 2.56 g/L after 24 h culture. These results were consistent with genomic transcript levels and strongly suggested that the mutation of *gltB* not only impaired the glutamate consumption significantly and but also decreased γ-PGA production of WT *B*. *subtilis* Bs916 sharply. In addition, our results also suggested the glutamate consumption and γ-PGA production showed a positive relationship.

**Table 5:** Glutamate consumption and γ-PGA production of Δ*gltB* mutant and WT *B*. *subtilis* Bs916.

Content (g/L)	Glutamate	γ-PGA
Bs916	2.68^a^	13.46^a^
Δ*gltB*	10.41^b^	2.56^b^
CK (EM)	20.0^c^	0^c^

a, b, and c mean in the same column with different letters are significantly different (P<0.05) according to Duncan’s multiple range tests.

### Biofilm restoration in the Δ*gltB* mutant

**Fig 6:**
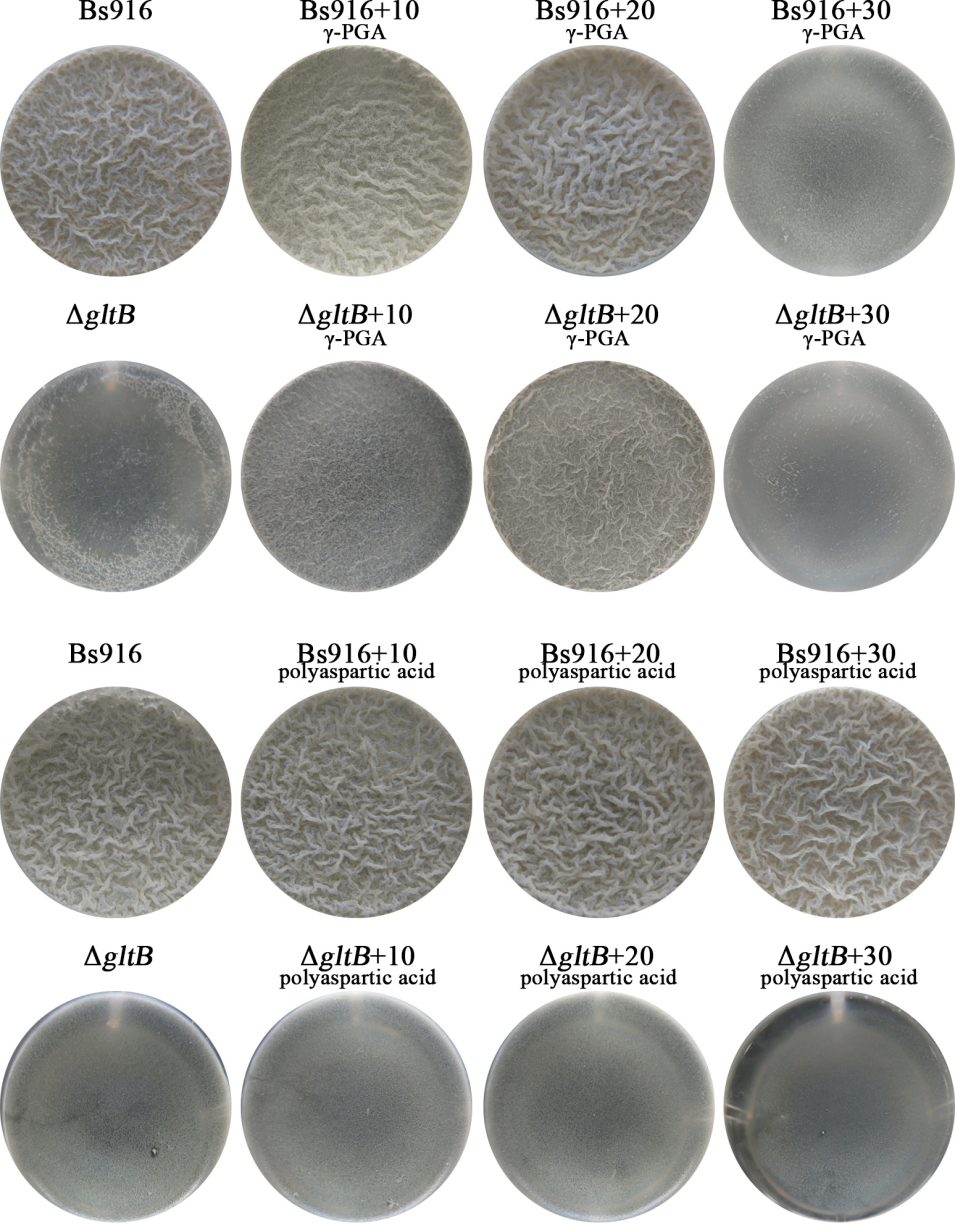
Biofilm restoration in the Δ*gltB* mutant. A final concentration of 10 g/L, 20 g/L, and 30 g/Lγ-PGA solution were added into the Δ*gltB* mutant in 4mL EM medium. The final concentration of 10 g/L and 20 g/Lγ-PGA were able to recover biofilm formation in the Δ*gltB* mutant, however, high final concentration of 30 g/Lγ-PGA inhibited biofilm formation in WT *B*. *subtilis* Bs916 and the Δ*gltB* mutant.

**Fig 7:**
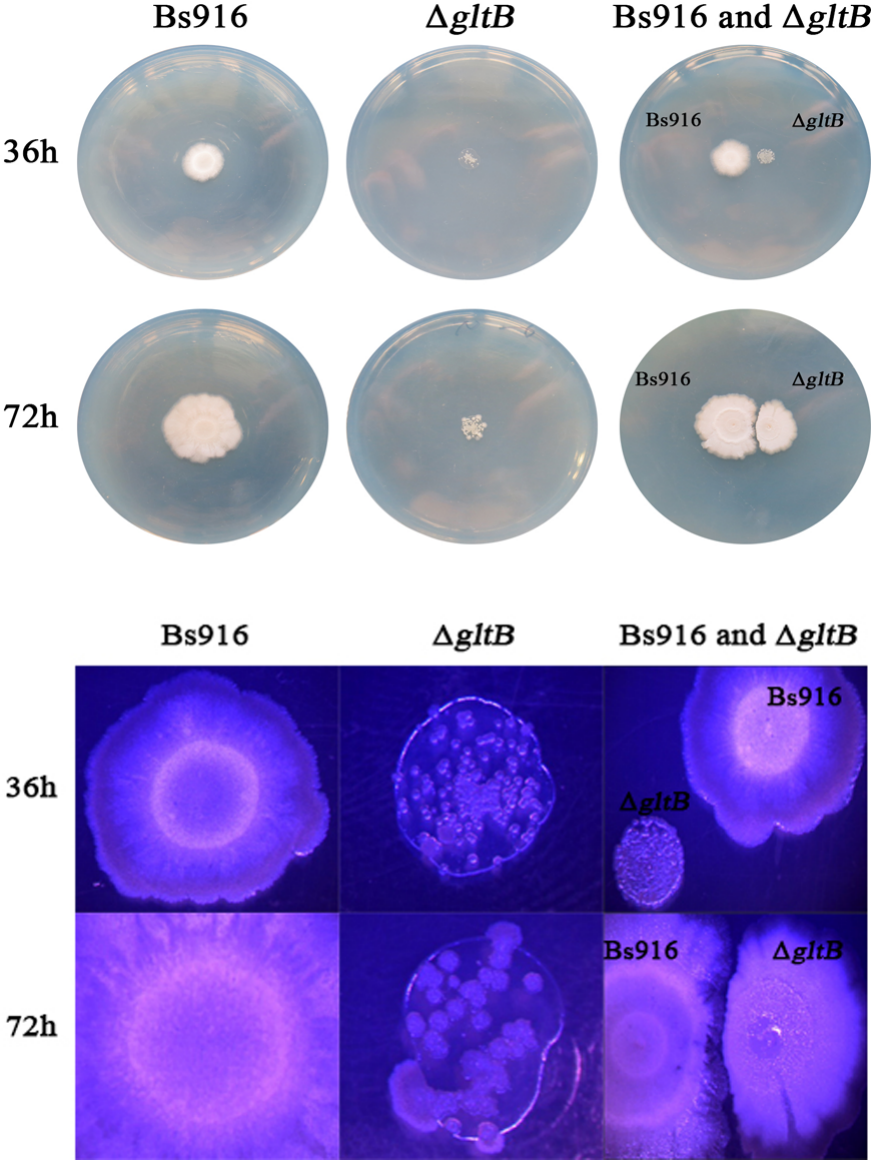
Biofilm restoration by *B*. *subtilis* Bs916’sγ-PGA in the Δ*gltB* mutant. The Δ*gltB* mutant grew next to *B*. *subtilis* Bs916 on solid EM plate with 1.5% agar to observe their biofilm formation. Biofilm formation of Δ*gltB* mutant was no significant restoration before 36 h. But in 72h, its biofilm formation had been restored to most parts.

Due to the sharply decrease in the production ofγ-PGA in the Δ*gltB* mutant, the restoration of biofilm by additionγ-PGA were investigated in detail. The results showed that the biofilm of Δ*gltB* was restored significantly both at the concentration of 10 g/L and 20 g/L of additionγ-PGA (Fig 6 and Table 6). Interestingly, when the concentration ofγ-PGA over 30 g/L, the biofilm formation of not only the Δ*gltB* mutant but also WT *B*. *subtilis* Bs916 were inhibited. This phenomena was also observed by Liu et al previously [18]. By plate confrontation growth for biofilm restoration in Δ*gltB* mutant, next to the WT *B*. *subtilis* Bs916 for 72 h, biofilm formation of Δ*gltB* mutant was no significant restoration before 36 h. But in 72h, biofilm formation had been restored to most parts (Fig 7). The control strain was only Δ*gltB* mutant, and its biofilm was still very poor. Taken together, we presumed that the loss of GltB inhibited biofilm formation of *B*. *subtilis* Bs916 maybe by altering the production of γ-PGA via the ability to consume glutamate.

**Table 6:** Biofilm dry weight analysis of Bs916 and Δ*gltB* mutant by adding γ-PGA and polyaspartic acid.

Strain	γ-PGA (g/L)				polyaspartic acid (g/L)			
	0	10	20	30	0	10	20	30
Bs916	10.5(0.3)	11.9(0.3)	12.8(0.5)	2.9(0.3)	9.9(0.3)	10.1(0.4)	10.4(0.4)	10.2(0.3)
Δ*gltB*	3.1(0.2)	4.7(0.3)	7.4(0.3)	2.7(0.2)	3(0.3)	3.1(0.3)	3.1(0.3)	2.9(0.3)

## Discussion

Rice sheath blight is a common fungal disease that affects rice globally, and it has resulted in more than a 15% production yield loss in China’s major rice growing areas [47–48]. In addition to the use of chemical pesticides to prevent the disease, *B*. *subtilis* has been widely used as a major biocontrol pesticide to prevent and control rice sheath blight [49–50]. The complete genome of *B*. *subtilis* Bs916 has been sequenced [33, 35], and sequence analyses along with detection of secondary metabolites showed that *B*. *subtilis* Bs916 was able to produce four LPs: bacillomycin L, surfactin, fengycin and locillomycin [34–35]. Here, we report a new and critical gene *gltB* which markedly affects biofilm formation, bacterial colonization and the biocontrol efficiency of *B*. *subtilis* Bs916 by regulating the production of γ-PGA and LPs.

The Δ*gltB* mutant showed serious flaws in its ability to form biofilms; the resulting biofilms were weak and thin with an irregular shape and a two-dimensional structure that differed greatly from the three-dimensional structure of the biofilms of the WT. To elucidate the regulatory mechanisms of *gltB* in biofilm formation, transcriptome analysis was used to identify differentially expressed genes between WT *B*. *subtilis* 916 and the Δ*gltB* mutant. The expression of *capB* (*ywsC*) was only one-fifth of the level of that in WT *B*. *subtilis* 916, and it was considered to control the biosynthesis of γ-PGA. Additionally, expression of the synthetic gene clusters for bacillomycin L, surfactin, and fengycin were also significantly altered. γ-PGA and the LPs are very important to biofilm formation [16–17, 39]. Therefore, we proposed that *gltB* regulates biofilm formation via a γ-PGA-dependent pathway and the production of the three LPs.

Our results strongly suggested that disruption of *gltB* resulted in impairment of the ability to assimilate glutamate and led to decreased γ-PGA biosynthesis. On the basis that γ-PGA was able to restore biofilm formation by the *gltB* mutant, we concluded that γ-PGA plays an important role in biofilm formation by *B*. *subtilis* 916. As we reported previously, surfactin was able to restore the biofilm formation of a bacillomycin L mutant [35]; therefore we think that the reduction of the bacillomycin L level in the Δ*gltB* mutant did not have a significant influence on biofilm formation by this strain because its increased production of surfactin would compensate for the decrease in bacillomycin L. In general, we confirmed that disruption of *gltB* led to deficiency in biofilm formation.

Colonization on rice surfaces and antifungal activity of *B*. *subtilis* 916 ensure its biocontrol efficiency against rice diseases. Biofilm formation is a prerequisite for bacterial colonization on plant surfaces and biocontrol efficiency [47, 51]. Bacillomycin L and fengycin are the main contributors to the antifungal activity of *B*. *subtilis* 916 [34–35]. Surfactin is main agent against bacterial pathogens such as *Pectobacterium carotovorum*, *Xanthomonas campestris* and *Podosphaera fusca* [48]. In our study, we conclusively proved that production of γ-PGA contributed to biofilm formation and verified that bacillomycin L and fengycin contributed to the antifungal activity of *B*. *subtilis* 916. Using confocal microscopy to detect colonisation of Δ*gltB* on rice plants and pot experiments to determinate its biocontrol efficiency, we showed that the Δ*gltB* mutant demonstrated poor colonisation, including fewer bacterial cells and a lack of significant nutrient chemotaxis. These results suggest that colonisation by *B*. *subtilis* Bs916 was regulated by GltB; it may also be influenced by the strength of the biofilm formation and the level of γ-PGA production [17]. The Δ*gltB* mutant also demonstrated poor biocontrol efficiency against rice sheath blight. So, we confirmed that biocontrol efficiency of *B*. *subtilis* Bs916 was regulated by GltB, mainly via the production of bacillomycin L and fengycin.

In conclusion, the production of γ-PGA contributes to biofilm formation. Bacillomycin L and fengycin contribute to the antifungal activity of *B*. *subtilis* 916 against *R*. *solani*. GltB regulates biofilm formation by altering the production of γ-PGA and the LPs bacillomycin L and fengycin, and influences bacterial colonisation of rice stems, which consequently leads to poor biocontrol efficiency of rice sheath blight.

## Supporting Information

S1 Fig Changes in colony architecture of the *B*. *subtilis* Bs916 and the Δ*gltB* mutant in MSgg culture medium with 20 μg/mL Congo Red and 10 μg/mL Coomassie brilliant blue. (PDF)
