# Supplementary material for: Loss of GltB Inhibits Biofilm Formation and Biocontrol Efficiency of Bacillus subtilis Bs916 by Altering the Production of γ-Polyglutamate and Three Lipopeptides
Source: PLoS One. 2016 May 25;11(5):e0156247. doi: 10.1371/journal.pone.0156247 (PMC4880196; doi:10.1371/journal.pone.0156247)

Supplementary Material

Loss of GltB inhibits biofilm formation and biocontrol efficiency of *Bacillus subtilis* Bs916 by altering the production of γ-polyglutamate and three lipopeptides

Plos one

Huafei Zhou[a,b], Chuping Luo[a,c], Xianwen Fang[b], Yaping Xiang[a,b], Xiaoyu Wang[b], Rongsheng Zhang[b], Zhiyi Chen[a,b]

([a] College of Plant Protection, Nanjing Agriculture Universtiy, Nanjing 210095，China;
[b] Institute of Plant Protection, Jiangsu Academy of Agricultural Sciences, Nanjing 210014，China;
[c] School of Life Science and Chemical Engineering, Huaiyin Institute of technology, Huaiyin 223003, China)

Address correspondence to Prof. Zhiyi Chen, 50 Zhongling Street, Nanjing, 210014, P. R. China, email: chzy84390393@163.com. Dr Huafei Zhou, 50 Zhongling Street, Nanjing, 210014, P. R. China, email: zhhf2010@126.com.

Supplementary Methods

Colony architecture observation of the *B. subtilis* Bs916 and the Δ*gltB* mutant by microscopy

To observe colony architecture, 1 μL of overnight broth culture was spotted onto the surface of an MSgg ager plate containing 20 μg/mL Congo Red and 10 μg/mL Coomassie brilliant blue, and incubated at 28 ℃ for 48 h (Romero *et al*. 2010, 2014). Colony architecture was observed by a 8–50 × objective dissecting microscope (Nuoxu-v JPX-200T, China). Each experiment was repeated three times.

Supplementary Figures

Fig. S1. Changes in colony architecture of the WT *B. subtilis* Bs916 and the Δ*gltB* mutant in MSgg culture medium with 20 μg/mL Congo Red and 10 μg/mL Coomassie brilliant blue.

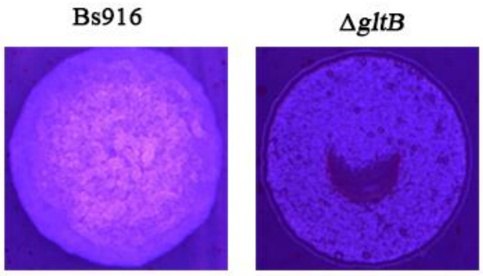

Supplement: S1 Fig — (PDF) [file pone.0156247.s001.pdf]
